# Application of Artificial Intelligence in Otology: Past, Present, and Future

**DOI:** 10.3390/jcm13247577

**Published:** 2024-12-13

**Authors:** Hajime Koyama, Akinori Kashio, Tatsuya Yamasoba

**Affiliations:** 1 Department of Otolaryngology and Head and Neck Surgery, Faculty of Medicine, University of Tokyo, Tokyo 113-8655, Japankashioa-tky@umin.ac.jp (A.K.); 2 Department of Otolaryngology, Tokyo Teishin Hospital, Tokyo 102-8798, Japan

**Keywords:** artificial intelligence, otology, generative AI, prediction, ChatGPT

## Abstract

Artificial Intelligence (AI) is a concept whose goal is to imitate human intellectual activity in computers. It emerged in the 1950s and has gone through three booms. We are in the third boom, and it will continue. Medical applications of AI include diagnosing otitis media from images of the eardrum, often outperforming human doctors. Temporal bone CT and MRI analyses also benefit from AI, with segmentation accuracy improved in anatomically significant structures or diagnostic accuracy improved in conditions such as otosclerosis and vestibular schwannoma. In treatment, AI predicts hearing outcomes for sudden sensorineural hearing loss and post-operative hearing outcomes for patients who have undergone tympanoplasty. AI helps patients with hearing aids hear in challenging situations, such as in noisy environments or when multiple people are speaking. It also provides fitting information to help improve hearing with hearing aids. AI also improves cochlear implant mapping and outcome prediction, even in cases of cochlear malformation. Future trends include generative AI, such as ChatGPT, which can provide medical advice and information, although its reliability and application in clinical settings requires further investigation.

## 1. Brief History of Artificial Intelligence

Artificial Intelligence (AI) is the concept or technique of imitating human intellectual activity in a computer. This has progressed rapidly in recent times, but its beginnings are not so long ago. In 1950, the concept of the Turing Test was proposed, and the word “artificial intelligence” first appeared in 1956 at the Dartmouth Conference.

AI has been studied since the 1950s, when it first appeared, and there have been three times when the study of AI boomed, and in-between the booms, such studies stagnated. Therefore, the study of AI has repeated this surge and stagnation. The current period is the third boom, and this boom will continue, and it is believed that we will not face stagnation again.

### 1.1. First Boom (Late 1950s to 1960s)

Since the appearance of the term, research on AI has spread rapidly. This was due to the fact that computers can automatically guess and find the answer to certain problems. In fact, the computer can solve simple tasks, such as a maze, but it was found that the computer could not handle complicated tasks when many factors are related to each other, and the research stagnated.

As for the neural network algorithm, which is the most important program in the recent development of artificial intelligence, it was also developed during this era. A neural network is a computational model that emulates the activity of a human neuron. The original concept of the artificial neuron was proposed by McCulloch and Pitts in 1943 [1]. This “formal neuron” is a simple model consisting of binary inputs and outputs, some restrictions on possible weights, and a more flexible threshold. In 1958, Rosenblat combined this formal neuron and created an artificial neural circuit, the perceptron, and introduced the idea of changing the weights to solve certain problems more efficiently [2]. This system received a lot of attention, but after it was shown that there were unsolved problems with the single-layer perceptron, the study languished.

### 1.2. Second Boom (1980s)

In the 1980s, researchers began to solve complicated tasks by adding some knowledge in advance. They tried to make the computer pretend to be an expert in decision making by giving the computer the expert’s knowledge transformed into the forms the computer could handle, called an expert system. In the medical field, the system called Mycin, which can diagnose the infection, is a representative of the system [3]. This system worked well in a certain field, but at that time the computer could not accumulate and update the knowledge. Therefore, humans had to provide all the knowledge needed to solve the problem to adapt it to the real world. In addition, there was a problem of how to deal with data when they conflicted with each other, and then the pace of research slowed down again in the 1990s.

Similarly, new breakthroughs were made in neural networks. At the end of the first boom, the single-layer perceptron proved to contain some unsolved problems, and to solve more complicated problems, the layers should be accumulated. However, the more layers that were accumulated, the more difficult was the weight to be determined. A new method, backpropagation, was proposed to determine the weights in multilayer neural networks [4]. It was possible to apply chain rules in the calculation of partial differentiation. This method is effective, but some problems were found. One of the major problems is called the vanishing gradient problem, which happens when the number of layers is increased, and the calculated gradient becomes small, and learning is less efficient. To overcome this problem, a new breakthrough is awaited.

### 1.3. Third Boom (From the Late 2000s to the Present)

The third expansion of research began in the late 2000s and continues to this day. There are several reasons why the research started to grow rapidly again, but the fact that the progress of computers increased the amount of data they can handle enabled researchers to use large amounts of data, big data, and led to the invention of the deep learning system. This is supported by an autoencoder system using the multilayer neural network developed by Hinton [5] and accounts for a large part of the third boom. The invention of the deep learning system made the computer learn by itself, and the advancement of computer technology realized the theoretically possible, driving the explosion of AI research.

Autoencoder is a neural network that extracts a key feature from the data. The autoencoder has two main components, which exchange the input data and produce the output data from the exchanged data. In the autoencoder, the input data is equal to the output data, and by comparing the difference between the input and output data, the autoencoder learns the appropriate change (encoding), which often works as a dimension reduction. What is important is that this process (learning or dimension reduction) is not controlled by human instruction or human designation. This process keeps humans away from providing the knowledge or condition, which makes a breakthrough and accelerates AI research.

At the same time, a new network model, the deep Boltzmann machine, has been proposed [6]. This method is an approximate approach for multi-layer network learning, which requires enormous computational overload, by applying learning to each layer and combining them. This approach turned out to provide reasonable approximate estimation in learning, allowing the network layer to be deeper.

The invention of the autoencoder and the deep Boltzmann machine helped the neural network to work in a deeper and more efficient way. These advances and the advancement of computer technology made it possible to handle a large amount of data, create deeper neural networks, and learn from these networks, realizing what was theoretically possible and driving the further explosion of AI research.

In particular, in 2012, the deep learning system beat other groups’ systems in the accuracy of the image recognition task at the ImageNet Large Scale Visual Recognition Challenge (IVLSC), attracting much attention in the field. This was followed by some landmark events, including Google’s invention of the system that could recognize the cat in the picture without human instruction, winning the champion in the Igo game, enabling the structural analysis of proteins [7], and winning novel prizes in various fields. Today, new algorithms and techniques are being developed every day, and research is expected to continue uninterrupted in the future.

## 2. The Terms “Artificial Intelligence”, “Machine Learning” and “Deep Learning”

There are several terms used to describe AI. AI has a variety of expressions. AI has the broadest meaning. AI is a general term used to describe computer systems or software that emulate the intelligence and behavior of human beings. Machine learning is a subfield of AI. Machine learning consists of three components; experience (E), task (T), and performance (P), and it lets a computer program learn from E, and learning means that T-measured P improves with the accumulation of E [8]. There are various algorithms used for learning such as naive Bayes, decision trees, random forests, the Markov model, the support vector machine, and neural networks.

Among these various algorithms, neural networks have been highly studied in recent years. When neural networks have many layers, the algorithms are called deep learning. Therefore, deep learning occupies a part of machine learning, but it has a broader ability to solve various problems by imitating human brain activity, which has led to a leading role in the third AI boom. A lot of studies have been done with the aim of clinical application.

## 3. Clinical Application in Otology

### 3.1. Diagnosis

#### 3.1.1. Otitis Media Diagnosis from Tympanic Membrane Analysis

Otitis media is a common disease in ENT, especially for pediatric patients. They suffer from hearing loss or delay in speech development if the effects continue. The sequelae sometimes last into adulthood [9].

There are some guidelines for the treatment of otitis media, and the evaluation of the tympanic membrane (TM) plays an important role in the guidelines. Despite the situation, it is sometimes difficult, especially for young physicians or physicians who do not specialize in otolaryngology, to correctly assess the condition of the TM, leading to an increasing demand for novel tools to diagnose the TM [10,11].

AI has been widely applied in many fields, but image analysis is one of the main areas of AI application [12]. The success of image classification was the epochal point where AI attracted attention again, and the boom of AI has continued since that event.

As a result, there are many reports on image analysis in the field of otolaryngology. A TM image is easy to obtain, and the number accumulates easily, so research has already been performed in deep learning analysis in the TM [13,14]. Many studies have applied transfer learning, applying the existing models, and the task has variety, such as pathology classification, detection of TM perforation, and tympanic tube detection. Each study has achieved about 80–90% accuracy, but the studies of physicians have been about 75% accurate, leading to the superiority of AI in accurate diagnosis [15]. Viscaino et al. used a support vector machine, k-nearest neighbor, and decision tree models to diagnose the three diseases, myringosclerosis, ear wax, and chronic otitis media. The SVM model using the filter-bank method [16] showed excellent results: 99.0%, 98.1%, 99.4%, and 98.14% in accuracy, sensitivity, specificity, and positive predictive values, respectively [17].

Livingstone et al. used the Google Cloud Vision AutoML system for the diagnosis and compared the accuracy with physicians from pediatrics, emergency medicine, otolaryngology, and family medicine. Overall, the average positive predictive value and the average sensitivity of AI was 90.9% and 86.1%, respectively, and the accuracy of AI was 88.7% compared with 58.9% for physicians [18]. Khan et al. applied one of the deep neural networks, DenseNet, for TM diagnosis, and reported 87% in classification accuracy compared with 74% in the average accuracy of 15 physicians, suggesting that the AI classification could be more of an accurate judgment [19]. Byun et al. applied a deep learning model, ResNet-18, in the classification of TM in comparison to doctors who had not majored in otolaryngology and residents, and the AI model superseded human diagnosis in terms of accuracy (97.1% vs. 82.9%) [20].

A direct comparison between physicians and the AI model showed that the accuracy of the machine was 95.5% and that of physicians was 65.0%, suggesting that the machine model can reduce misdiagnosis, inappropriate antibiotic use, and unnecessary surgery [21]. Continuous research is performed to improve the accuracy, and Zefer et al. achieved high accuracy, 99.5%, in differentiated diagnosis of acute otitis media, otitis media with effusion, and chronic purulent otitis media by fusing fine-tuned deep features of models [22].

The TM images themselves comprise a variety of types, from otoscopic images to photos taken by a smartphone. Some research suggests that smartphone images could be an appropriate object for deep learning analysis, and machine models have shown high accuracy, 90.7%, in diagnosis with learning from TM images from smartphones, suggesting the robustness to the quality of these images [23]. Furthermore, not only photos but also videos could be objects of analysis. A protocol has been reported that extracts some images from video recording of the TM and creates images to facilitate the detection of the pathological area [24].

In this way, TM analysis by AI is possible, and it could achieve higher accuracy than that of clinicians’ analysis. In addition, images captured by smartphones can be useful, suggesting that this system would be useful in remote areas where few otologists work or in daily examinations performed by patients themselves. However, there is still some need to acquire TM images, and the accuracy will deteriorate if some earwax remains obstructing the TM images. In addition, otitis media is a common disease, especially in pediatric patients, but some cases occur where children cannot be cooperative in taking TM images, which reduces the usefulness of this system. These systems are theoretically possible, but they have been kept from clinical use, and these challenges should be overcome.

#### 3.1.2. Temporal Bone CT Analysis

In addition to TM images, temporal bone CT is a key component in the diagnosis and treatment of otologic disorders. It plays an important role, not only in the diagnosis of disease, but also in preoperative planning and landmark identification in the perioperative period. Recent advances have been made in the analysis of temporal CT images using deep learning techniques.

There have been several reports on the segmentation system using AI for normal temporal CT images. Several networks, including the anisotropic hybrid network, U-Net, residual neural network [25], W-Net [26,27], PWD-3DNet, a patch-wise network from DensVNet [28], have been applied to detect anatomically important areas such as middle ear ossicles, the labyrinth, or the facial nerve. Ding et al. reported comprehensive segmentation and detection of the malleus, incus, stapes, labyrinth, and facial nerve, in submillimeter accuracy, using the new U-Net, an open source 3-dimensional semantic segmentation neural network [29].

Some methods were used to perform segmentation automatically, and other methods aimed to improve accuracy. In general, to improve the precision of detection, several steps that humans enact, such as post-processing, are required, and much effort has been made to achieve these two ambivalent goals at the same time.

In addition, many previous studies have used 3D convolutional models to analyze consecutive temporal CT images. This is a reasonable approach because CT images have a 3D structure. However, the computation in 3D models takes a long time to learn, and some studies use 2D models instead of 3D models and achieve similar accuracy [30,31,32].

In addition to segmentation, the diagnosis of the pathology of ear disease in CT images has been performed using AI. Fujima et al. reported the diagnosis of otosclerosis on temporal bone CT using deep learning [33]. They applied AlexNet, VGGNet, GoogLeNet, and ResNet to 198 temporal bone CT images and compared the accuracy of diagnosis between the models and the radiologist, and reported that there was no difference between the corresponding models and the radiologist. The diagnostic accuracies were 0.89, 0.72, 0.81, 0.86, and 0.86 for the subspecialty-trained radiologist, AlexNet, VGGNet, GoogLeNet, and ResNet, respectively. There were no significant differences in performance between AI models, such as VGGNet, GoogLeNet, and ResNet, and the radiologist. GoogLeNet and ResNet, furthermore, showed non-inferiority compared to the radiologist. Li et al. reported the use of deep learning in the diagnosis of cochlear malformation in temporal bone CT and its superiority in accuracy compared with the diagnosis of the chief physician [34]. They applied four network model, ResNet10, ResNet50, SE-ResNet50, and DenseNet121, and the reported positive predictive values were 0.82, 0.83, 0.85, and 0.71, respectively. Three of them were higher than the highest positive predictive value of the human otology doctor and chief physicians (0.8). Wang et al. reported the diagnosis program between normal, chronic otitis media, and cholesteatoma using a deep learning model, Inception-V3 [35]. They used 1147 ears as a dataset and reported an accuracy of 76.7%, which was higher than the average of six clinical experts at 73.8%. Takahashi et al. used MobileNetV2 and detected cholesteatoma extending to the mastoid cavity in 164 patients [36], and Eroglu et al. further extended the study by combining deep learning image analysis by Alexnet, Googlenet, and Densenet201 and support vector machine classification to diagnose chronic otitis media and cholesteatoma [37]. They achieved high accuracy (95.4%) with these combinations, and this could be a promising tool for the diagnosis of temporal bone CT.

#### 3.1.3. Brain MRI Analysis

MRI also plays an important role in decision-making in clinical otology. It is also becoming the object of AI, but there is a smaller number of studies than CT.

Vestibular schwannoma is the most common target in the application of AI to MRI. MRI is the high-priority recommendation to detect the vestibular schwannoma [38] and the inner ear consistency in appearance compared with the schwannoma, which increases the importance and ease in the application of AI techniques for tumor detection and diagnosis. Shapey et al. reported that the segmentation of the machine model was as accurate as that of experts [39]. They developed a novel AI framework based on a 2.5D neural network and reported 93.7%, 0.20, and 7.03% for the Dice score, which reflects the similarity of the two groups, average symmetric surface distance, and relative volume error, respectively. They concluded considering a margin of 5% for the Dice score, and there was no statistic difference between AI segmentation and professional radiologist segmentation. Neve et al. invented an automatic segmentation system using the data of 214 cases from 37 institutions in the multicenter study [40] and reported accurate detection. Neves et al. developed a deep learning model using 490 adult patients with vestibular schwannoma to delineate the tumor and inner ear and tested the accuracy using an open public dataset (Vestibular Schwannoma SEG). They reported high accuracy with mean errors of predicted tumor volume, tumor centroid location, and inner ear centroid location of 0.05 cm3, 0.52 mm, and 0.85 mm, respectively. Not only the solid portion, but also the cystic area was detected by the AI model using the U-net convolutional neural network [41].

In general, the models created by AI depend on the training data, and if the data were collected in a single center or a using specific scanner and acquisition protocol, then the reliability of the other images inconsistent with the training data decreases. Therefore, the models should be trained using data from multiple institutions and a wide range of protocols to increase robustness for daily clinical use. Kujawa et al. used images from the routine clinical dataset of 10 institutions and invented the segmentation system without relying on a specific acquisition protocol [42]. They applied a neural network, U-Net, and the reported Dice similarity coefficients were 95.5 in Gamma Knife stereotactic radiosurgery, and 86.4 in the multi-center routine clinical dataset. They achieved a similar result as radiologists in segmentation, not only for specific image datasets obtained for Gamma Knife stereotactic radiosurgery, but also for multicenter routine clinical datasets, while previous models created from specific image datasets did not show inadequate performance in the daily use dataset, suggesting the importance of the diversity of training datasets.

In addition to vestibular schwannoma, other diseases have been investigated using AI. Other research focused on measuring endolymphatic hydrops in patients with Meniere’s disease. Cho et al. created a convolutional neural network model to perform automatic segmentation of the cochlea and vestibule and calculate the ratio of endolymphatic hydrops [43]. They compared the calculated ratio of endolymphatic hydrops from AI and from a neuro-otologist and neuro-radiologist and reported the intraclass correlation was 0.97, concluding the consistency of the result obtained by the machine model with the figure estimated by the neuro-otologist and neuro-radiologist. Park et al. updated the system and increased the coherence between the machine-based figure and the manually measured figure [44]. They also reported that it took about 3.5 seconds for the developed program to correctly analyze the endolymphatic result, which led to the rapid provision of objective analysis results and reduced the workload of physicians.

### 3.2. Treatment

#### 3.2.1. Systemic Steroids for Sudden Sensorineural Hearing Loss

Sudden sensorineural hearing loss (SSNHL) is a common condition with an incidence of no less than 5–27 per 1,000,000 [45]. It is often defined as a hearing threshold elevation of at least 30 dB at three consecutive frequencies on a pure-tone audiogram [46,47].

Systemic steroids are used to treat SSNHL [48]. There is little scientific evidence for its efficacy [49,50,51], but because SSNHL causes severe symptoms, the AAO-HNS guideline recommends the use of systemic steroids and it is widely used worldwide [51]. Many studies have been conducted on hearing prognosis and hearing improvement after the treatment of SSNHL. Moreover, age [52], concomitant symptoms such as vertigo and tinnitus [53], duration from onset to treatment [54], severity [55], hearing loss pattern [56], and complications [57,58] have been reported as prognostic factors [59]. Despite these studies, it has been difficult to predict the hearing outcome after treatment for SSNHL. Therefore, AI has been applied to predict hearing recovery in SSNHL.

Lee et al. analyzed a total of 453 unilateral SSNHL patients using logistic regression (baseline), a support vector machine, extreme gradient boosting, light gradient boosting, and a multilayer perceptron, and concluded that the light gradient boosting machine was the best. They examined the machine learning model using the SHapley additive explanation and reported that the initial audiogram shape was the most important prognostic factor [60]. Another article applied ensemble learning models based on boosting, bagging, AdaBoost, and stacking algorithms in 1572 SSNHL patients and reported that the AdaBoost algorithm achieved more than 80% accuracy and precision (accuracy 82.9%, precision 86.7%) for predicting hearing outcome and found that age and time to treatment were the most significant prognostic factors [61].

Considering SSNHL associated with vertigo, a report analyzed the findings in vHIT by using a convolutional neural network of coherent frequency with high magnitude-squared wavelet coherence with wavelet coherence analysis, and elucidated the accurate prediction of hearing outcomes [62].

There are several similar studies reporting that the random forest algorithm was the best [63] or the deep belief network was the most accurate predictor, and there is no consensus on the best algorithms [63]. They compare several techniques, including the deep belief network, together with conventional logistic regression, the support vector machine, and multilayer perceptron. They concluded that the deep belief model achieved the highest accuracy and area under the curve, with results of 77.6% and 0.84, respectively. Aghakhani et al. [32] in their review article reported seven studies using machine learning techniques in predicting hearing outcomes after treatment of SSNHL and they pointed out the limitation that all these studies did not accept the external validation and the risk of bias was high, and they did not apply the study outcome in the external world. Treatment controls, data collection methods, and other factors, including physician quality, are different between institutions, and the dataset used in these studies could be biased. Multicenter data collection could be helpful to reduce these biases, and prospective validation of the developed model in different institutions where the dataset was collected will increase the reliability of these models. Unfortunately, to date, there is no research on external validation of the prediction models created. They also commented that all of these studies were conducted in China or Korea, and there may be regional differences that require additional research to apply the result of the model to the real world [32].

#### 3.2.2. Tympanoplasty

Tympanoplasty is the main surgical procedure for otitis media. It can increase the hearing level with the cessation of otorrhea, but the improvement of postoperative hearing varies among patients, and it remains difficult to predict the postoperative hearing level before surgery.

Szaleniec applied k-nearest neighbor and neural network algorithms to patients with chronic otitis media to predict hearing outcomes after tympanoplasty and reported 84% correct predictions of whether or not the postoperative hearing level was better than the preoperative hearing level [64]. Koyama et al. applied a machine learning technique to predict hearing outcomes and identify prognostic factors in a patient undergoing tympanoplasty for chronic otitis media [65]. They applied three algorithms, the random forest, support vector machine, and k nearest neighbor, and compared the prediction accuracy with conventional scoring predictions. They reported that the random forest algorithm achieved 63.1% in accuracy in multiclass classification compared to 28.9% in the conventional model and concluded the superiority of machine learning over the classical scoring system. Lim et al. studied 484 patients with chronic otitis media who underwent tympanoplasty and they evaluated several AI models and reported that preoperative hearing status was the most important factor [66]. They applied the light gradient boosting machine and multilayer perceptron models as AI models, and logistic regression was used for the base line model. They also reported that the gradient boosting machine exhibited a significantly higher area under the curve compared to that of the baseline model (mean, 0.811). AI would be an appropriate approach for predicting outcomes in otologic surgery, but there are still several factors, such as the volume of datasets, diversity of patient backgrounds, and surgical techniques that hinder the progress of research, and only a limited number of studies have been reported so far.

#### 3.2.3. Hearing Aids

Hearing aids (HAs) are used for patients with hearing loss, and for much mild to moderate hearing loss, HAs are the only approach for treatment [67]. HAs themselves are beneficial, but they have limitations for patients in restoring “normal” hearing. Patients tend to be less satisfied, especially in difficult conditions for hearing, such as noisy environments and environments with multiple speakers. Some reports have used deep learning methods to reduce noise in HAs and have reported improvement in hearing with HAs [68,69,70] or by separating competing voices [71]. Recent research suggests that a deep learning-based HA noise reduction system improves hearing clarity in hearing impaired patients [72]. For example, hearing aids have multiple modes that correspond to a particular sound environment, such as music, and AI can detect the situation and change the mode in real time, resulting in improved patient satisfaction [73]. In addition, some AI features, such as Starkey’s Edge Mode, can take a “snapshot” of the sound environment based on the user’s instruction and can optimize the setting according to the environment [74].

Fitting is also important for the patient to achieve satisfactory hearing with HAs. Traditionally, fitting is performed in two steps, fitting by formulas and fitting by an audiologist according to the hearing level [75,76]. However, a recent study suggested that the application of a machine learning technique in the fitting process could achieve better results than the company’s autofitting and could improve the gap between the initial fitting and the appropriate fitting, leading to better fitting satisfaction [77].

#### 3.2.4. Cochlear Implant

Cochlear implant (CI) is the treatment for patients with severe to profound hearing loss and it can improve their hearing and speech perception. AI has been used in the field of cochlear implantation.

Recent CI processors have adopted AI to optimize the hearing quality by changing the settings, such as automatic sensitivity control, dynamic range optimization, and directional changes [78,79]. Sahoo et al. reported that these AI-based speech processors improve patients’ satisfaction and signal-to-noise ratio [80].

Although CI is an effective treatment for patients, the hearing outcome varies from patient to patient, and it is difficult to know the postoperative outcome preoperatively. Some research has been performed to solve the problem and predict the hearing outcome with CI. Guerra-Jiméne et al. applied several machine learning algorithms such as nearest neighbor and decision tree algorithms [81]. Crowson et al. reported CI outcome prediction with an accuracy of 95.4% in the classification task using a neural network [82]. Even for the patients with inner ear malformation, Weng et al. applied the k-nearest neighbor algorithm and achieved an accuracy of 93.3% and 86.7% in the classification task of hearing rehabilitation and speech rehabilitation, respectively [83]. Lu et al. reported CI outcome prediction for cochlear nerve deficiency patients with 71% accuracy and 0.71 AUC in predicting hearing rehabilitation and 93% accuracy and 0.94 AUC in predicting speech rehabilitation using a support vector machine [84]. This expansion of AI applications could lead to a general prediction model for CI outcomes with different backgrounds.

CI mapping is also a target of AI applications as well as HA fitting. Meeuws et al. invented computer-assisted mapping software using AI [85], and Waltzman et al. reported a similar result of AI mapping to conventional mapping [86]. Koyama et al. reported a better postoperative T level prediction of pediatric patients by AI and better prediction accuracy compared with classical statistical analysis [87]. A recent study suggested that an AI-assisted CI mapping tool could improve the audiological outcomes of CI patients [88], and AI could help both audiologists and patients enjoy satisfactory mapping and better hearing outcomes in less time than the current approach.

## 4. Challenges

Despite the rapid progress of AI, there are still some unresolved issues regarding the application of AI in healthcare. Preparation of high-quality training data, robustness of image variations, and management of uncooperative patients are some of the challenges facing most current AI systems. These should be addressed in future studies.

Previous AI studies have relied heavily on annotated datasets (supervised learning), and this has shown great success at the laboratory level. However, in order to apply this AI in the real world, it is necessary to increase the validity and reliability of the data not involved in model building. To improve such external validity, one key is that AI has learned from huge amounts of data, but medical data labeling requires specialists and is cumbersome, leading to a decrease in the amount of data. Therefore, semi-supervised or self-supervised learning plays an important role in the process of improving AI for clinical use [89]. Multimodal learning, which refers to learning from a variety of modalities, such as numbers, images, sounds, is one choice to combat these problems. ChatGPT 4 can handle not only text but also images and is an example of multimodal AI. New model architecture, known as transformers and used in ChatGPT, was introduced by Vaswani and colleagues in 2017 [90] and it enables AI to handle multimodal datasets, leading to the analysis of large amounts of data without fully annotated inputs and ground truths (semi-supervised/unsupervised learning) and the creation of robust AI.

Because of these unresolved issues, despite these advantages of AI, all studies on outcome prediction for otologic diseases or treatments, such as SSNHL or cochlear implants, are still at the proof-of-concept stage and not at the real-world clinical stage [91]. There is no commercial base application of AI in otology outcome prediction, which should be solved as soon as possible.

## 5. Ethical Concerns

With the progress of AI, there are several concerns about ethical, legal, or privacy issues. When AI is applied to clinical medicine, you need to pay attention to several factors that involve the use of AI.

### 5.1. Algorithmic Ethics

#### 5.1.1. Bias

There are several bias issues in the use of AI. One is data bias, which refers to the presence of biases in the training data when a particular AI has been developed. Castro et al. classified bias into five patterns: population shift, prevalence shift, acquisition shift, annotation shift, and manifestation shift [92]. Population shift occurs when the datasets have specific characteristics such as age, ethnicity, or habits that could be correlated with the judgment. Prevalence shift occurs when there is an unbalanced prevalence in the datasets. Acquisition shift is the most common bias in medical image analysis. There are discrepancies between institutions in how medical images are acquired, such as radiation dose level, slice thickness, and kernel. These differences in dataset acquisition suffer from acquisition bias. Annotation shift occurs especially when people annotate the dataset. In the manual segmentation, there is often a decrease in interobserver variability. Manifestation sift is the difference of appearance in the same clinical diagnosis. There is a wide variety of clinical manifestations even in one disease such as chronic otitis media, so it could become a cause of bias in the dataset.

Even if there are no unbalanced data in the dataset, algorithms themselves contain bias. This is called algorithmic bias, and it can come from biased feature selection, biased model design, or biased decision rules [93]. This could lead to incorrect suggestions or decisions by the AI.

#### 5.1.2. Reliability and Transparency

Because AI may make incorrect decisions, the reliability of AI is crucial. To ensure or increase the reliability of AI, it is important to achieve transparency in AI. Transparency refers to how the AI works and makes the final judgment in the system [94]. AI secured transparency is called explainable AI, and some methods are proposed to ensure transparency, such as local interpretable model-agnostic explanations, and visualization, such as Google DeepDream [95]. This explainability is especially important in generative AI, such as ChatGPT, so it would be necessary to show how to generate outputs and use them in clinical applications.

### 5.2. Legal Ethics

#### 5.2.1. Responsibility

The application of AI output in clinical use raises concerns about legal responsibility, given the potential for AI misjudgment. In short, who will bear responsibility for medical errors resulting from AI? There is not enough case law or guidelines on this, but so far, from a legal perspective, AI lacks legal status, and humans must be the ultimate duty bearers [96]. Of course, human oversight is needed to enjoy the benefits of AI output, and ChatGPT recommends that users seek appropriate advice from specialists. However, this ambiguity about responsibility hinders the facilitation of AI use, and clear regulations or guidelines are needed to encourage the use of AI in clinical settings.

#### 5.2.2. Privacy

Since AI requires a lot of data to generate the output, how to collect big data or whether or not privacy is protected in the collection process is very important. There is a concern about the possibility of unauthorized access or processing in data collection. In the medical field, the data include race, social history, medical history, images, diagnosis, and so on, which is highly sensitive information. Regulations, such as the Health Insurance Portability and Accountability Act (HIPAA) in the United States, play an important role in the use of AI to protect privacy [97], and the provider and researcher must comply with these regulations not only to protect patient privacy, but also to facilitate research.

In addition, natural language processing, such as ChatGPT, can cause other privacy issues. These models have the possibility of inadvertently disclosing sensitive information or harming patient privacy or well-being through inaccurate responses [98]. Regular monitoring and auditing are necessary to use this technique in daily clinical practice.

## 6. Future Perspective

Since the advent of AI, models have been developed to address or solve specific problems. However, new generative AI without a specific purpose has attracted much attention since the appearance of ChatGPT version 4 (Open AI, San Francisco, CA, USA) in 2023. ChatGPT is a generative AI that helps users obtain appropriate answers when they ask. It can respond to medical questions such as “What is the best treatment for chronic otitis media?” or “How to treat tinnitus and what causes it?” It can search for clinical evidence or articles that users want to know about and suggest them in a very short time, but because of the short time that has passed since ChatGPT became available, the reliability of the response has not been fully investigated and there is little consensus on how to handle it.

Frosolini assessed the reliability of ChatGPT by reviewing the literature and found modest success in the field of otolaryngology [99]. Aliyeva et al. reported on the efficacy of ChatGPT in supporting CI patients. They reported that ChatGPT could provide the appropriate answer to the frequently asked question about postoperative care of CI in a few seconds and concluded that it could be helpful, especially for patients [100]. On the other hand, Maksimoski et al. concluded that the correct answer rate for questions related to otolaryngology topics was 45.5%, and the rate became worse when the question related to clinical guidelines, highlighting the limitations of generative AI [101]. The advantages and disadvantages of generative AI, such as ChatGPT, have not been fully investigated, and more research is needed in this area.

AI often takes a long time to learn, but it takes little time to generate the outputs of its learned algorithms. This is a useful point in busy clinical environment. For example, AI can reduce the time of writing the report of a radiological image by 50%, and physicians could receive the reports of AI immediately [12]. We could identify the pathological area while taking the otoscopic image or give the predicted outcome of SSNHL as soon as the hearing test is received by integrating the AI system into audiometry. We could give the predicted postoperative outcome at once by entering the key clinical features in some system such as apps. There could be a near future where AI systems are integrated into the daily routine in some specific clinical settings.

AI could integrate other technologies, such as augmented reality or wearable devices. AI can segment disease in image analysis and display it in augmented reality. This could be helpful not only for surgeons in preoperative preparation or teaching young doctors but also for patients in recognizing their disease or how they would be operated upon. The wearable device is a good tool for AI analysis, because the wearable device can collect patients’ data in real-time, which helps to collect a lot of information that AI needs for analysis, leading to the increased reliability and validity of AI. In addition, the instancy wearable device could be helpful for patient care because it can make immediate feedback possible. Some otological diseases, such as hearing loss or dizziness, occur rapidly, and acute intervention is essential for treatment. Wearable devices and instant analysis of AI would be beneficial for patient care. Other diseases, such as Meniere disease, are often affected by environmental conditions, including atmospheric pressure and humidity [102], and wearable devices accumulate this information and could offer preventable approaches. A wearable device, such as smart glass, can display captions of the difficult parts for hearing impaired patients as soon as they hear a voice.

## 7. Conclusions

AI research is expanding rapidly, and many topics are becoming targets. In otology, not only the diagnosis process but also the prediction of treatment is being investigated using AI. Image analysis has been the main target of AI, and great progress has been made, but now there is an increasing number of studies on clinical outcome prediction. In addition, new generative AI, such as ChatGPT, has emerged with the ability to change clinical processes. The reliability of the response has not been fully verified, and this reliability mainly depends on the information used to feed AI software, but as research progresses, it could drastically change the process of both routine medical care and research investigation.

## Data Availability

The data that support the findings of this study are available from the corresponding author, upon reasonable request.
